# Molecular Detection of Extended‐Spectrum Beta‐Lactamase (ESBL) Genes in *Escherichia coli* Isolated From Vegetables and Environmental Samples in Selected Urban Farms and Wet Markets in Metro Manila, Philippines

**DOI:** 10.1155/ijfo/6681719

**Published:** 2026-04-20

**Authors:** Donnabel C. Sena, Ma. Christine Jasmine F. Sabio, Pierangeli G. Vital

**Affiliations:** ^1^ Natural Sciences Research Institute, University of the Philippines Diliman, Quezon City, 1101, Philippines, upd.edu.ph

## Abstract

*Escherichia coli* (*E. coli*) is a facultative anaerobic Gram‐negative bacterium that is not only commensal, inhabiting the intestinal tract of humans and warm‐blooded animals, but also a major opportunistic pathogen causing several diseases, mainly foodborne. *E. coli* is also involved in a wide range of niches including plant surfaces and tissues. Some strains of *E. coli* produce extended‐spectrum β‐lactamases (ESBL), which reduces the efficacy of β‐lactam antibiotics which is one of the most commonly used antibiotics. Therefore, contamination of fresh produce with *E. coli*, potentially carrying ESBL, can pose a significant health risk. This study aims to detect ESBL genes in *E. coli* isolated from raw vegetables, using in‐house designed primers for polymerase chain reaction (PCR). A total of 117 fresh produce and environmental samples was obtained from selected urban gardens, along with 348 fresh produce from selected wet markets in Metro Manila, Philippines. Using culture in mTEC agar and confirmation by *uidA* gene detection using conventional PCR, a total of 74 samples tested positive for *E. coli*. These were subjected to phenotypic and molecular detection of ESBL genes, using primer pairs for three ESBL genes *blaCTX-M*, *blaTEM*, and *blaSHV* designed in this study. Overall, the prevalence of ESBL‐producing *E. coli* in this study was 5.41%. The primers designed successfully amplified the desired amplicon sizes, demonstrating potential utility in PCR‐based detection of ESBL genes in *E. coli* isolates. The results of the research may be used to improve the current detection methods of ESBL‐producing bacteria thereby contributing to the current knowledge on antimicrobial resistance in foodborne pathogens.

## 1. Introduction

*Escherichia coli* (*E. coli*) is a facultative, anaerobic Gram‐negative bacterium that inhabits the intestinal tracts of humans and warm‐blooded animals. While most *E. coli* strains are commensals, they retain remarkable genetic flexibility that enables them to adapt to a diverse niche and acquire certain virulence traits. As a result, *E. coli* is not only a natural resident of the gut but also a major opportunistic pathogen associated with diarrheal diseases, urinary tract infections, septicemia, neonatal meningitis, and other clinical infections in both humans and animals [1, 2]. Indeed, it is recognized as one of the most significant foodborne pathogens alongside *Salmonella* spp. and *Campylobacter* [2]. *E. coli* is a common contaminant in food production systems, particularly in fresh produce. Vegetables can become contaminated at multiple points along the farm‐to‐fork chain, including through irrigation water, soil, fertilizers, animal intrusion, handling by farm workers, and postharvest processing [3, 4]. Vegetable contamination by organisms, such as *E. coli*, can happen through various mechanisms. *E. coli* has been shown to attach to root surfaces whereby it can be internalized into the plant shoots and other tissues. Contamination of stems and vegetables also happens via the stomata, necrotic lesions, or through the damaged tissues of the vegetables and further proliferates through biofilm formation [4]. Numerous foodborne outbreaks linked to leafy greens and other fresh produce highlight the public health concern surrounding this organism [5].

*E. coli* also plays a critical role in the global antimicrobial resistance (AMR) crisis. Lacking intrinsic resistance to most antimicrobials, *E. coli* is highly receptive to selective pressures imposed by antimicrobial use in humans and food‐producing animals [6]. The presence of antimicrobial‐resistant *E. coli* in fresh produce, especially the extended‐spectrum β‐lactamase (ESBL)‐producing strains, additionally represents a food safety issue that can potentially cause both agricultural and clinical risks [5, 7].

ESBLs are a diverse group of β‐lactamases that are characteristically able to hydrolyze third‐generation cephalosporins as well as aztreonam but can be inhibited by β‐lactamase inhibitors, such as clavulanic acid [8, 9]. ESBL‐producing microorganisms not only complicate treatment with the use of β‐lactam antibiotics but they can also potentially complicate the treatments with other drugs as reports show that the presence of ESBLs can co‐occur with other antibiotic resistance genes conferring resistance to antibiotics, such as colistin, carbapenem, aminoglycosides, and fluoroquinolones [10–12]. Additionally, this concerns both the healthcare industry and the agricultural sector due to transmissibility [13]. ESBL‐producing *E. coli* (ESBL‐*E. coli*) had been observed to be transmitted between humans, livestock, and food crops, as well as through the environment [13–19]. Several reports from the Philippines highlight the presence of ESBL‐*E. coli* from both agricultural and clinical settings. Vital et al. [19] and Gundran et al. [20] demonstrated the presence of ESBLs found in irrigation water and broiler farms, respectively. This highlights the risk of cross‐contamination between animal hosts and fresh produce. However, ESBL‐*E. coli* have been demonstrated in hospital isolates by Cruz et al. [21] and Lota and Latorre [22] wherein both studies show the alarming increase in the presence of ESBL‐producing bacteria isolated from humans.

ESBLs are a significant factor that affects the effectiveness of antibiotic treatment [8–12]. There is a significant amount of literature showing the presence of ESBL‐*E. coli* in fresh produce [7, 23–25]. In recent years, there has been an increase in the preference for the consumption of raw or minimally processed fresh produce due to nutritional advantages [26, 27]. Thus, surveillance of ESBLs is needed to help prevent the further spread of antibiotic resistance and ensure food safety.

Currently, conventional phenotypic methods, such as the combination disk test, double disk synergism test, and ESBL brilliance agar screening [28], have been the cornerstone in the surveillance of antibiotic resistance. However, the main disadvantage of the phenotypic method is the long turnaround time which can delay the decision‐making process for the proper course of antibiotic treatment. Fortunately, with the advancement of technology, detection of AMR genes, such as ESBL, can now be carried out using nucleic acid amplification assays targeting ESBL‐associated genes, such as *blaSHV*, *blaTEM*, and *blaCTX*‐*M* [29]. Molecular methods, such as the polymerase chain reaction (PCR), provide a faster way of detecting and identifying pathogenic species, virulence determinants, and AMR genes, therefore overcoming the disadvantages brought about by the phenotypic methods.

This study aims to apply the PCR‐based method in the detection of ESBL genes present in *E. coli* isolates obtained from selected urban farms and wet markets in Metro Manila, Philippines. Specifically, the researchers aim to develop new primer sets to be used in the detection of ESBL genes.

## 2. Materials and Methods

### 2.1. Sample Collection and Isolation of *E. coli*

Three urban farms and four wet markets in Metro Manila, Philippines, were selected as sampling sites for this study. Vegetable species and environmental samples (i.e., soil, irrigation water, and domestic animal feces) were randomly collected based on their availability at each location. These samples were collected and processed immediately in the laboratory for *E. coli* isolation using mTEC agar (Millipore, USA), a medium used to selectively grow thermotolerant *E. coli*, directly from wash samples without the need for prior enrichment steps. Then, confirmation of *E. coli* was performed molecularly, through the detection of the *uidA* gene in the isolates by conventional PCR. A more detailed description of the sampling, *E. coli* isolation, and *E. coli* confirmation was discussed in [30]. Confirmed *E. coli* isolates were maintained in a microcentrifuge tube of 1‐mL trypticase soy broth (TSB) (BD, Germany) with 30% glycerol for long‐term storage.

### 2.2. ESBL Screening and Confirmation (Phenotypic)

*E. coli* isolates in glycerol stocks were first revived before ESBL screening. Briefly, the glycerol stocks were centrifuged at 10,000 rpm, and the supernatant was discarded. The pellet was resuspended using 200 μL of fresh TSB, and using a sterile loop, it was streaked onto nutrient agar plate and incubated at 37°C for 24 h. To ensure that the isolates were free of contaminants, they were restreaked to Eosin Methylene Blue (EMB) (BD, Germany) agar plates and incubated at 37°C for 24 h for the observation of the typical *E. coli* colonies (green to black colonies with or without the presence of green metallic sheen). The cultures were then screened phenotypically for ESBL production. Colonies from the EMBA plates were picked and transferred to 0.85% NaCl solution until a turbidity equivalent to that of 0.5 McFarland solution was achieved. A sterile cotton swab was dipped in the solution and used to make a lawn on Mueller–Hinton Agar (BD BBL) plates containing the following 30 μg antibiotic disks: ceftazidime, cefotaxime, ceftriaxone, and aztreonam (Oxoid). The plates were incubated at 35°C for 18 h. The zones of inhibition were measured, and samples that showed a diameter of less than 22 mm for ceftazidime, 27 mm for aztreonam, 25 mm for ceftriaxone, and 27 mm for cefotaxime were suspected to be ESBL producers (Table 1).

**TABLE 1:** Zone diameter interpretative as potential ESBL‐producing *E. coli* based on CLSI M100 performance standards for antimicrobial susceptibility testing, 34^th^ edition [31].

Antibiotic	Zone of inhibition (mm)
Ceftazidime (CAZ)	≤ 22
Aztreonam (ATM)	≤ 27
Ceftriaxone (CRO)	≤ 25
Cefotaxime (CTX)	≤ 27

**FIGURE 1:**
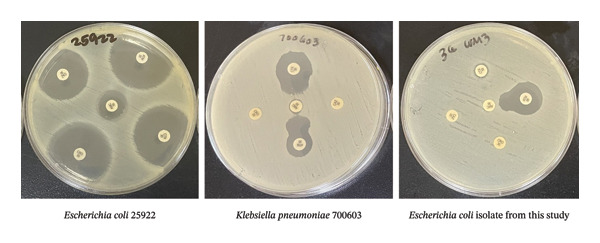
DDST showing the characteristic zone of inhibition that is enhanced toward the clavulanic acid–containing disk, also called the “keyhole” zone of inhibition pattern.

Presumptive ESBL producers were then subjected to phenotypic confirmation of ESBL according to the methods of Kumar et al. [32], done using the double disk synergy test (DDST). Disks containing 30 μg of ceftazidime, cefotaxime, ceftriaxone, and aztreonam were placed on an MHA plate with amoxicillin/clavulanate disk placed on the center of the plate. If the zone of inhibition was observed to be pointing toward the direction of the amoxicillin–clavulanate antibiotic disk, it was considered an ESBL producer (Figure 1). *E. coli* 25,922 and *Klebsiella pneumoniae* 700,603 were used for the negative and positive controls, respectively.

### 2.3. ESBL Gene Primer Design

The National Center for Biotechnology Information (NCBI) database was queried for three ESBL genes (*blaCTX-M*, *blaTEM*, and *blaSHV*), and the first returned sequences for each gene were used (accession numbers: MT636767.1 for *blaCTX-M*, KU664682.1 for *blaTEM*, and NG_148673.1 for *blaSHV*). Using the sequences obtained, primers were designed using NCBI Primer Blast where default parameters were used except as follows: (1) minimum, optimum, and maximum primer melting temperatures were 55, 58, and 60, respectively; (2) database was set to “nr,” and (3) primers must have at least 3 total mismatches to unintended targets and at least 3 mismatches within the last 5 bps at the 3′ end. Then, using Primer Stat (bioinformatics.org), GC clamp, hairpin, and self‐annealing were analyzed to check for primer stability. The primer sequence was sent to Macrogen Asia Pacific Pte Ltd for synthesis.

### 2.4. Molecular Confirmation of ESBL Genes

Overnight cultures in tryptic soy broth (Merck) were subjected to DNA extraction using NEB Monarch Genomic Extraction Kit, following instructions. PCR amplification of ESBL genes was done using (1) the primers from published literature using the PCR settings described in each reference and (2) using the newly designed primer in this study (Table 2) and the PCR settings described in Table 3. The PCR amplicons were visualized using gel electrophoresis using the following settings: For amplicons below 200 bp, gel electrophoresis was run using 2% agarose gel for 35 min at 100 V. The 50 bp DNA ladder (Cleaver) was used for checking the size of bands. For amplicons 200 bp and larger, gel electrophoresis was run using 1% gel for 35 min at 100 V and using a 1 kb ladder (Meridian Bioscience 1 kb HyperLadder).

**TABLE 2:** Primers used in this study.

Primer name	Sequence (5′‐3′ direction)	Reference
CTX‐M‐U1	5′‐ATGTGCAGYACCAGTAARGTKATGGC‐3′	[33]
CTX‐M‐U2	5′‐TGGGTRAARTARGTSACCAGAAYCAGCGG‐3′	[33]
TEM‐F	5′‐GCG​GAA​CCC​CTA​TTT​G‐3′	[34]
TEM‐R	5′‐ACC​AAT​GCT​TAA​TCA​GTG​AG‐3′	[34]
blaSHVF	5′‐ATG​CGT​TAT​ATT​CGC​CTG​TG‐3′	[35]
blaSHVR	5′‐TGC​TTT​GTT​ATT​CGG​GCC​AA‐3′	[35]
CTX‐M750F	5′‐GGG​TAA​AGC​ATT​GGG​TGA​CA‐3′	this study
CTX‐M154R	5′‐GAT​ATC​GTT​GGT​GGT​GCC​AT‐3′	this study
TEM870F	5′‐TAA​CTC​GCC​TTG​ATC​GTT​GG‐3′	this study
TEM324R	5′‐GAC​TCC​CCG​TCG​TGT​AGA​TA‐3′	this study
SHV861F	5′‐CGC​CAT​TAC​CAT​GAG​CGA​TA‐3′	this study
SHV419R	5′‐CCC​GCA​GAT​AAA​TCA​CCA​CA‐3′	this study

**TABLE 3:** Polymerase chain reaction conditions used for amplification of ESBL genes.

Steps	Temperature (°C)			Duration (seconds)		
	CTX‐M750F/CTX‐M154R	TEM870F/TEM324R	SHV861F/SHV419R	CTX‐M750F/CTX‐M154R	TEM870F/TEM324R	SHV861F/SHV419R
Initial denaturation	95	95	95	180	180	180
Denaturation	95	95	95	60	60	60
Annealing	59	59	58	30	30	30
Extension	72	72	72	20	30	30
Final extension	72	72	72	300	300	300
Number of cycles	20					

## 3. Results

A total of 117 fresh produce and environmental samples from selected urban gardens and 348 fresh produce from selected wet markets were obtained, with a total of 74 isolates molecularly confirmed to be *E. coli* (Table 4). On the 74 *E. coli* isolates, phenotypic detection of ESBL was performed using the DDST, which showed that 4 of 74 isolates were positive for the presence of ESBL (5.41%) (Table 5). Using the primers published by earlier authors, ESBL genes *blaCTX-M* and *blaTEM* were detected in all the 4 positive samples, while *blaSHV* was not detected. The same results were obtained using the primers developed in this study (Figures 2, 3, and 4). Appropriate results were also obtained when tested against *E. coli* ATCC 25922, 2 nontarget bacteria, 3 phenotypically negative *E. coli* isolates from this study, and the recommended positive controls for the ESBL genes (*Klebsiella quasipneumoniae* ATCC 700603 and *Salmonella* spp.). This suggests that the developed primers are sensitive only to the intended targets, although more study must be done on this to include more samples (Table 6).

**TABLE 4:** Prevalence of *E. coli* in various sample matrices collected from urban gardens and wet markets in Metro Manila.

Sample matrix	Prevalence of *E. coli*	
	Wet market	Urban farms
Soil	—	2/18 (11.11%)
Fecal samples from domestic animals	—	10/11 (90.91%)
Fresh produce	42/348 (12.07%)	15/71 (21.13%)
Irrigation water	—	5/17 (29.41)

**TABLE 5:** Confirmation of ESBL in *E. coli* isolates from vegetables through DDST and PCR (*N* = 75).

Isolate ID	DDST result				PCR result		
	AZT	CTX	CAZ	CRO	*blaCTX-M*	*blaTEM*	*blaSHV*
36WM3	+	+	−	−	+	+	−
1UG2W	+	+	+	+	+	+	−
F2UG2W	+	+	+	−	+	+	−
F2UG1W	+	+	+	+	+	+	−

FIGURE 2Detection of *blaCTX-M* gene using primers CTX‐M‐U1/CTX‐M‐U2 (a) and CTX‐M750F/CTX‐M154R (b). The primers successfully amplified the target *blaCTX-M* gene of around 593 and 154 bp. (samples from lane 2: positive control, negative control, phenotypically ESBL‐positive *E. coli* isolates).

**Figure:**
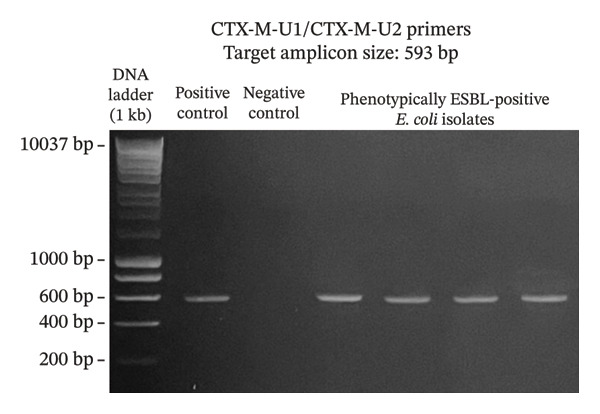
(a)

**Figure:**
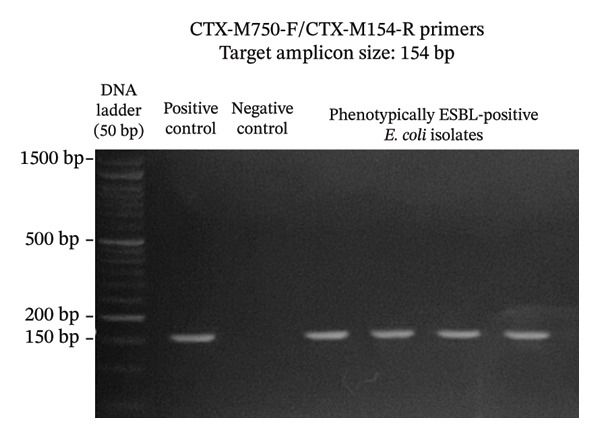
(b)

FIGURE 3Detection of *blaTEM* gene using primers TEM‐F/TEM‐R (a) and TEM870F/TEM324R (b). The primers successfully amplified the target *blaTEM* gene of around 964 and 324 bp. (samples from lane 2: positive control, negative control, phenotypically ESBL‐positive *E. coli* isolates).

**Figure:**
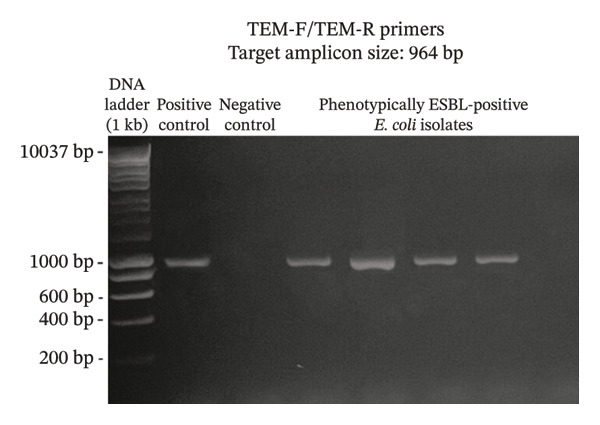
(a)

**Figure:**
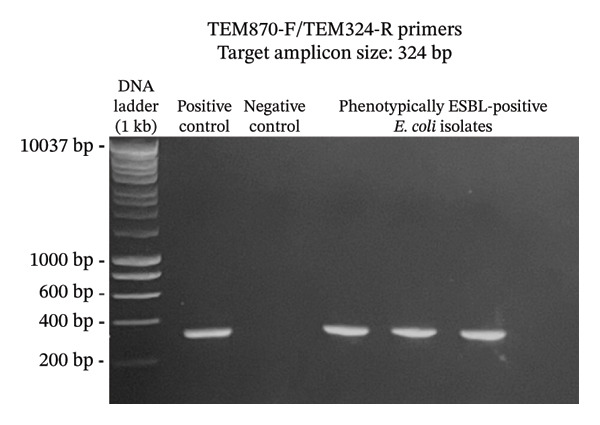
(b)

FIGURE 4Detection of *blaSHV* gene using primers blaSHVF/blaSHVR (a) and SHV861F/SHV419R (b). The primers successfully amplified the target *blaSHV* gene of around 745 and 419 bp. (samples from lane 2: positive control, negative control, phenotypically ESBL‐positive *E. coli* isolates).

**Figure:**
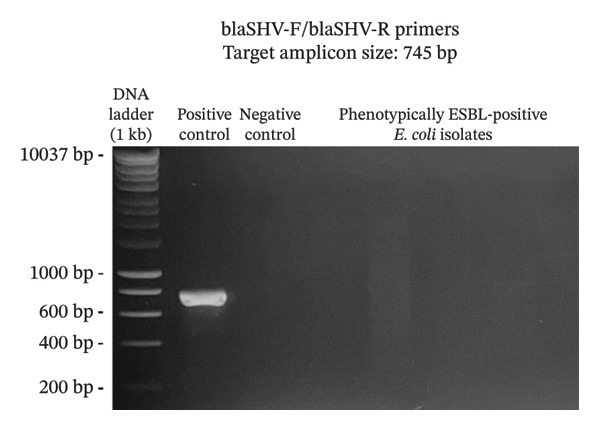
(a)

**Figure:**
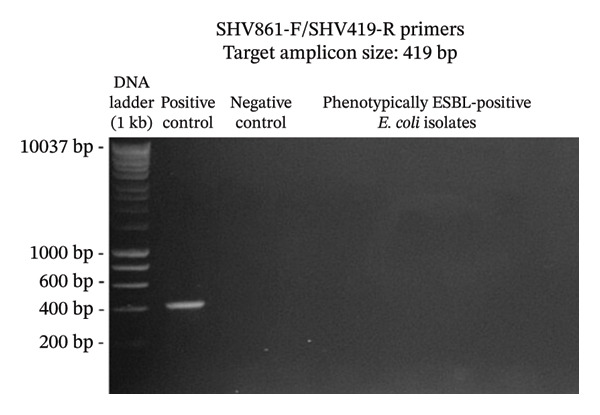
(b)

**TABLE 6:** Characteristics of the primers designed in this study.

Primer name	Product length	tm	%GC	GC clamp	Self‐annealing	Hairpin formation
CTX‐M750F	154	60.50	50	Pass	Pass	Pass
CTX‐M154R		59.50	50	Pass	Pass	Pass

TEM870F	324	59.50	50	Pass	Pass	Pass
TEM324R		61.00	55	Pass	Pass	Pass

SHV861F	419	58.6	50	Pass	Pass	Pass
SHV419R		59.9	50	Pass	Pass	Pass

## 4. Discussion

Our results demonstrated a low prevalence of ESBL‐*E. coli* (5.33%), similar to reports from South Korea (0.83%, *N* = 1324) and Thailand (4.6%, *N* = 305) [24, 25]. Additionally, the ESBL‐*E. coli* from the South Korean study was also found to be multidrug resistant (MDR), notably to ampicillin, nalidixic acid, piperacillin, cefazoline, and cefotaxime [24, 25]. However, a relatively higher prevalence has been reported in the region. In a study conducted in Malaysia, more than half of the samples of lettuce (*N* = 95) and bean sprouts (*N* = 85) were found to harbor ESBL‐*E. coli* [7]. In the study done in Pakistan, nearly half of *E. coli* isolates were phenotypically ESBL‐positive [36]. These findings highlight the widespread contamination of vegetables with *E. coli* and the presence of ESBL‐producing strains, indicating potential health risks associated with the consumption of raw or minimally cooked vegetables, despite the low prevalence observed. However, direct comparisons should be interpreted with caution due to methodological and contextual differences. This includes variations in sample size, samples analyzed, sampling strategies, and season, as well as detection techniques used. These factors can substantially influence the reported prevalence. For instance, leafy vegetables with larger surface areas may be prone to contamination (Murtaza et al., 2023), while differences in cultivation, irrigation, and postharvest practices may further contribute to variability. These considerations highlight the need for standardized methodologies in assessing *E. coli* as well as the presence of antibiotic‐resistant strains in fresh produce.

The presence of ESBL genes confers resistance to antibiotics, such as penicillin, cephalosporins (first to third generation), and aztreonam [37]. These antibiotics are important to both human health and agriculture, as they are the most prescribed antibiotics to treat infections caused by *Listeria*, *Neisseria*, *Proteus mirabilis*, *Salmonella*, *Shigella*, and *E. coli*, among others [38]. ESBL resistance leads to the compromised or prolonged treatment of these infections, resulting in significant medical and agricultural loss. Additionally, resistance to ESBLs is often associated with resistance to other common antimicrobial agents used clinically, such as sulfamethoxazole, gentamicin, trimethoprim, and fluoroquinolones [39]. Furthermore, bacterial plasmids of ESBL‐producing *Enterobacteriaceae* (ESBL‐E) may also harbor plasmids that carry a few other antibiotic resistance genes [40, 41]. For example, a study demonstrated the cotransfer of fluoroquinolone resistance determinant with ESBL‐carrying plasmids [42, 43]. This further extends resistance to multiple classes of antibiotics, making treatment more challenging. Additionally, this leads to even heavier usage of a variety of antibiotics which is also another risk factor in further acquisition of antibiotic resistance determinants. In fact, through the years, there has been an increased trend in antibiotic resistance to the preferred antibiotics including third‐generation cephalosporins, ampicillin, gentamicin, and tetracycline [44]. Therefore, ESBL producers pose a significant challenge in the clinical setting.

Despite the low occurrence observed in this study, the detection of ESBL genes in both vegetable and environmental samples is noteworthy. Environmental matrices, such as soil, irrigation water, and domestic animal feces, are important reservoirs of AMR. Contaminated irrigation water particularly sourced from untreated surface waters can introduce ESBL‐ and other antibiotic‐resistant bacteria directly onto crops (Igbinosa et al., 2023). Similarly, soil may serve as a long‐term reservoir for resistant organisms, especially when exposed to runoff or untreated manure. The detection of ESBL‐E in this study supports the hypothesis that fresh produce contamination may occur preharvest through environmental pathways. In addition, the presence of domestic animals in or near urban farming systems may further contribute to fecal contamination, facilitating the transfer for resistant bacteria to soil, and therefore, crops (Devarajan et al., 2023).

While phenotypic detection of bacteria via culture is very important in its surveillance and, therefore, control, this method is laborious and time‐consuming which warrants a need to develop and optimize methods for fast detection. Detection of pathogens via PCR has been explored in the literature and applied in the field. For example, when the *uidA* gene has been established to be present in about 94%–96% of *E. coli* strains, it has been the focus of primer design and application for its molecular detection [45, 46]. However, the *uidA* primer has its shortcomings, such as producing false‐positive results involving *Hafnia alvei* and *Serratia* spp. instead of *E. coli*. In addition, another primer called the *lacZ*, only correctly identified 70% of the 324 coliform isolates [47]. Primers for various other gene targets for *E. coli* were designed and are widely used in the literature, such as the *yaiO* [48], the *ECO* [49], and *uspA* [50].

Aside from the identification of pathogenic bacteria, primer design studies have also focused on the detection of antibiotic resistance genes in bacteria, and these proved advantageous as well for understanding the dissemination of these genes to other organisms as well as the environment. However, there are a few studies that conducted primer design and validation for the detection of ESBL genes in *E. coli*. A key contribution of this study is the design and preliminary validation of primers targeting three clinically relevant ESBL genes (*blaCTX-M*, *blaTEM*, and *blaSHV*)*.* Analysis using PrimerStat showed that the primers passed primer stability parameters, such as the percent GC content, self‐complementarity in the 5′ and 4′ ends, GC clamp formation, self‐annealing, and hairpin formation. The performance of these primer pairs was compared with the previous ESBL gene primers described in the literature (Figures 2, 3, and 4) using in vitro PCR amplification. Although the new set of primers targeted different regions of the selected genes and thus resulted in different amplicon sizes, they showed parallel results with the previous primers used. There were amplifications on the expected band size for both *blaCTX-M* and *blaTEM*, while the desired bands for *blaSHV* were not detected for the *E. coli* isolates in this study. Unlike conventional phenotypic methods, this gene‐targeted PCR approach enables direct detection of resistance determinants, allowing for more specific and sensitive identification of ESBL‐producing organisms. This is particularly valuable for detecting low‐abundance or nonculturable bacteria and for understanding the genetic basis of resistance dissemination in environmental and food matrices. Furthermore, the primers demonstrated stable amplification and similar melting temperatures, supporting their potential application in a multiplex PCR format. To further validate the utility of these newly developed primers, a wider scale field application must be done to generate results based on a larger sample size. The next step is also to develop a multiplex PCR using these primers as their melting temperatures are almost identical.

This study demonstrated the presence of ESBL genes from *E. coli* isolated from raw vegetables from selected supermarkets and urban gardens in Metro Manila, Philippines. As there is an increasing popularity of eating minimally processed vegetables due to nutritional benefits, this indicates a potential threat of consumption of ESBL‐*E. coli*‐contaminated raw vegetables. Further, aside from the immediate health consequences, ESBL‐*E. coli* can also colonize the gastrointestinal tract, and over the last 3 years, the human gastrointestinal carriage of ESBL‐*E. coli* was seen to be uprising, as shown in a meta‐analysis by Bezabih et al. [51]. This can potentially lead to shedding and dissemination of ESBL‐*E. coli* to the environment through domestic waste drainage onto receiving rivers or other surface waters [52].

Future research should focus on several key areas. First, quantitative risk assessments are needed to better estimate the public health impact of ESBL contamination in fresh produce. Second, further validation of the designed primers using a broader range of isolates and sequencing confirmation is necessary to establish their robustness and specificity. Third, the development and optimization of a multiplex PCR assay based on these primers would enhance their applicability in routine surveillance and outbreak investigations. Additionally, future studies should investigate the relative contribution of different environmental sources to contamination pathways through source‐tracking approaches. Longitudinal monitoring of urban farming systems would also provide insights into temporal trends and the effectiveness of intervention strategies. Finally, integrating molecular tools with environmental and epidemiological data will be critical for developing targeted control measures to mitigate the spread of ESBL‐producing bacteria along the farm‐to‐fork continuum.

## 5. Conclusions

This study showed ESBL genes in the *E. coli* isolates from selected urban gardens and wet markets in Metro Manila and provided data on the possible utility of newly designed primers for the molecular detection of ESBL genes. However, this is just a preliminary study, and further study must be done to ensure the efficiency of these primers. It is also highly recommended to use these primers for a wider scale survey of ESBL‐*E. coli* using a larger number of samples as well as on sample types other than raw vegetables (i.e., environmental samples, clinical samples, or meat and dairy products). Furthermore, application of these primers on a multiplex PCR can also be tried in the future for a faster detection of ESBL genes.

## Funding

This work was supported by the University of the Philippines Diliman Office of the Vice Chancellor for Research and Development (OVCRD) through Outright Research Grant under grant number 232327 ORG.

## Conflicts of Interest

The authors declare no conflicts of interest.

## Data Availability

The data that support the findings of this study are available from the corresponding author upon reasonable request.
